# A Novel Tri-Hydroxy-Methylated Chalcone Isolated from *Chromolaena tacotana* with Anti-Cancer Potential Targeting Pro-Survival Proteins

**DOI:** 10.3390/ijms242015185

**Published:** 2023-10-14

**Authors:** Gina Mendez-Callejas, Marco Piñeros-Avila, Juvenal Yosa-Reyes, Roberto Pestana-Nobles, Ruben Torrenegra, María F. Camargo-Ubate, Andrea E. Bello-Castro, Crispin A. Celis

**Affiliations:** 1 Grupo de Investigaciones Biomédicas y de Genética Humana Aplicada (GIBGA), Laboratorio de Biología Celular y Molecular, Facultad de Ciencias de la Salud, Universidad de Ciencias Aplicadas y Ambientales (U.D.C.A), Calle 222 # 55-37, Bogotá 111166, Colombia; marpineros@udca.edu.co; 2 Grupo de Investigación en Ciencias Exactas, Física y Naturales Aplicadas, Facultad de Ciencias Básicas y Biomédicas, Laboratorio de Simulación Molecular y Bioinformática, Universidad Simón Bolívar, Carrera 59 # 59-65, Barranquilla 080002, Colombia; juvenal.yosa@unisimon.edu.co (J.Y.-R.);; 3 Grupo de Investigación en Productos Naturales de la U.D.C.A. (PRONAUDCA), Laboratorio de Productos Naturales, Universidad de Ciencias Aplicadas y Ambientales (U.D.C.A), Calle 222 # 55-37, Bogotá 111166, Colombia; 4 Grupo de Investigación en Fitoquímica (GIFUJ), Departamento de Química, Facultad de Ciencias, Pontificia Universidad Javeriana, Cra. 7 # 40-62, Bogotá 1115511, Colombia

**Keywords:** *Chromolaena tacotana*, hydroxy-chalcone, cancer cells, intrinsic apoptosis, autophagy

## Abstract

*Chromolaena tacotana* (*Klatt*) R. M. King and H. Rob (*Ch. tacotana*) contains bioactive flavonoids that may have antioxidant and/or anti-cancer properties. This study investigated the potential anti-cancer properties of a newly identified chalcone isolated from the inflorescences of the plant *Chromolaena tacotana* (*Klatt*) R. M. King and H. Rob (*Ch. tacotana*). The chalcone structure was determined using HPLC/MS (QTOF), UV, and NMR spectroscopy. The compound cytotoxicity and selectivity were evaluated on prostate, cervical, and breast cancer cell lines using the MTT assay. Apoptosis and autophagy induction were assessed through flow cytometry by detecting annexin V/7-AAD, active Casp3/7, and LC3B proteins. These results were supported by Western blot analysis. Mitochondrial effects on membrane potential, as well as levels of pro- and anti-apoptotic proteins were analyzed using flow cytometry, fluorescent microscopy, and Western blot analysis specifically on a triple-negative breast cancer (TNBC) cell line. Furthermore, molecular docking (MD) and molecular dynamics (MD) simulations were performed to evaluate the interaction between the compounds and pro-survival proteins. The compound identified as 2′,3,4-trihydroxy-4′,6′-dimethoxy chalcone inhibited the cancer cell line proliferation and induced apoptosis and autophagy. MDA-MB-231, a TNBC cell line, exhibited the highest sensitivity to the compound with good selectivity. This activity was associated with the regulation of mitochondrial membrane potential, activation of the pro-apoptotic proteins, and reduction of anti-apoptotic proteins, thereby triggering the intrinsic apoptotic pathway. The chalcone consistently interacted with anti-apoptotic proteins, particularly the Bcl-2 protein, throughout the simulation period. However, there was a noticeable conformational shift observed with the negative autophagy regulator mTOR protein. Future studies should focus on the molecular mechanisms underlying the anti-cancer potential of the new chalcone and other flavonoids from *Ch. tacotana*, particularly against predominant cancer cell types.

## 1. Introduction

**Figure 1:**
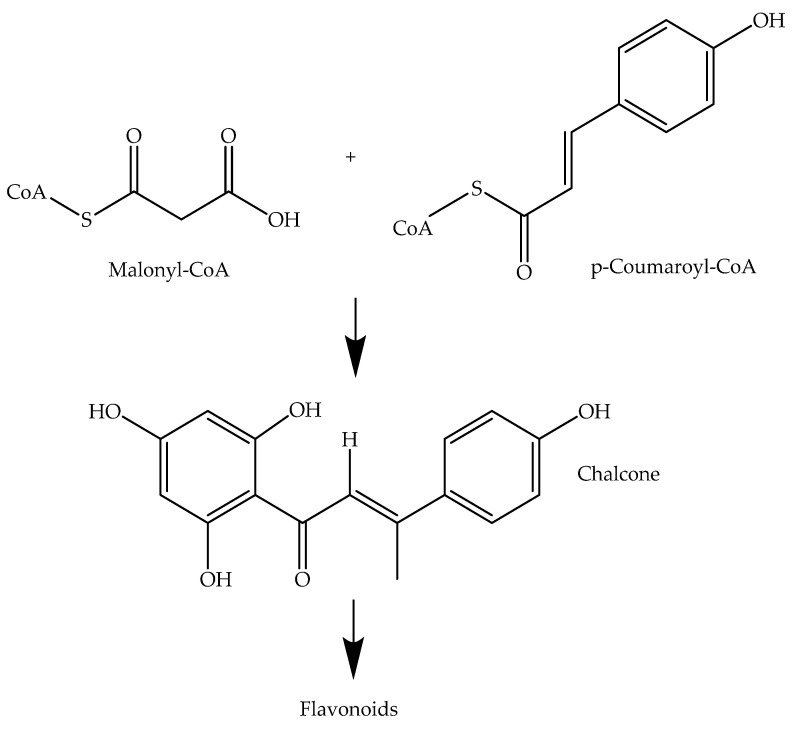
Chalcones as intermediates of the flavonoid biosynthetic pathway.

Cancer, one of the leading causes of death worldwide, necessitates continuous improvement in treatment options [1]. Flavonoids, bioactive polyphenols with various therapeutic potentials, including anti-cancer activity, have been extensively studied in both preclinical and clinical settings [2]. The natural biosynthesis of flavonoids involves the activity of chalcone synthase, which utilizes p-coumaroyl CoA and malonyl CoA as precursors to catalyze the formation of a hydroxy-α- and β-unsaturated ketone (1,3-diaryl-2-propen-1-one). This intermediate serves as a precursor to a wide range of flavonoids [3] (Figure 1).

Chalcones are widely distributed in fruits, vegetables, spices, tea, and soy-based foods [4,5]. However, their low accumulation levels make isolation and purification challenging, leading to the synthesis of derivatives as a common practice [6]. Due to their unique structure [7], chalcones have gained prominence for their various therapeutic properties, including their potential as anti-cancer agents [8]. In fact, some chalcones have already undergone clinical trials for cancer treatment [6].

Several natural chalcones with anti-cancer potential have been reported [1,9]. However, only a few of these natural chalcones possess both hydroxy and methoxy groups in their structures (Table 1). One well-known subgroup of these chalcones is called flavokavains, which were originally identified in *Piper methysticum* (*Kava*) [10,11].

The Chromolaena genus has a history of being used in traditional medicine for treating diseases [12,13]. Among the bioactive compounds found in Chromolaena species, flavonoids have demonstrated antioxidant and anti-cancer properties [14,15,16].

Both *Chromolaena chasea* and *Chromolaena odorata* have been reported to possess anti-cancer potential due to the presence of different polyphenolic compounds, including hydroxy-methylated chalcones such as ChDora and Odoratin [14,17,18,19]. ChDora has shown cytotoxic activity against glioblastoma cell lines [20], while Odoratin has demonstrated cytotoxicity against breast and cervical cancer cells [14].

**Table 1:** Natural hydroxy-methoxy chalcones with anti-cancer properties.

Chalcone	Plant Source	Type of Cancer	Reference
Helichrysetin	*Alpinia (Zingiberaceae)* and *Helichrysum (Asteraceae)*	Cervical, gastric, glioblastoma, and lung	[21,22,23,24,25]
Xanthohumol	*Humulus lupulus (Cannabaceae)*	Melanoma, gastric, breast, prostate, lung, pancreatic, ovarian, neuroblastoma, prostate, and colon	[26,27,28,29,30,31,32,33].
Cardamonin	*Zingiberaceae* family, *Artemisia* and *Helichrysum (Asteraceae)*, *Carya (Ju-glandaceae)*, *Vitex (Verbenaceae)*, *Desmos(Annonaceae)*, *Comptonia* and *Morella (Myricaceae)*, *Combretum (Com-bretaceae)*, *Syzygium (Myrtaceae)*, *Piper (Piperaceae)*, *Polygonum (Polygona-ceae)*, *Populus (Salicaceae)*, *Cedrelopsis (Rutaceae)*, and *Woodsia (Dryopteridaceae)*	Breast, gastric, colon, and ovarian	[34,35,36,37,38,39,40,41,42]
Flavokawain A	*Piper methysticum (Piperaceae)* and *Goniothalamus gardneri (Annonaceae)*	Breast, lung, and prostate	[1,43,44,45,46]
Flavokawain B	*Piper methysticum (Piperaceae)*, *Alpinia pricei Hayata*, and *Alpinia pricei rhizome (Zingiberaceae)*	Breast, colon, lung, and prostate	[1,47,48,49,50,51,52,53,54]
Flavokawain C	*Didymocarpus corchorijolia (Gesneriaceae)* and *Aniba riparia (Lauraceae)*	Breast, colon, and ovarian	[1,55,56,57,58,59]
Licochalcone A	*Glycyrrhiza glabra* and *Glycyrrhiza inflata (Fabaceae)*	Colon, breast, lung, and bladder	[60,61,62,63,64,65,66,67,68,69,70,71]
Broussochalcone A	*Broussonetia papyrifera (Moraceae)*	Renal	[72]
Isobavachalcone	*Psoralea corylifolia (Leguminosae)*	Breast	[73,74]
Pinostrobin	*Uvaria chamae (Annonaceae)*	Breast and prostate	[75,76]

*Chromolaena tacotana* is an endemic plant in Colombia. Its leaves contain flavonoids that have exhibited cytotoxic activity against various cancer cell lines [15,16,20]. Additionally, a flavanone previously identified in the plant’s leaves has shown the ability to induce cell death through apoptosis in triple-negative breast cancer (TNBC) cells, further supporting its anti-cancer potential [16]. This study aims to report the chemical characterization and selective anti-cancer potential of a recently identified tri-hydroxy-di-methoxy chalcone isolated from the inflorescences of *Ch. tacotana* on predominant types of cancer cell lines.

## 2. Results

The chalcone was isolated from the dichloromethane inflorescence extract of *Ch. tacotana.* Its chemical structure was elucidated through the analysis of ^1^H and ^13^C Nuclear Magnetic Resonance (NMR) experiments and High-Resolution Mass Spectrometry (MS). The UV spectra were also analyzed with displacement reagents such as AcONa, MeONa, AlCl_3_/HCl, and H_3_BO_3_.

### 2.1. Structural Analysis

The chalcone was obtained as an orange powdery solid soluble in acetone, with melting point (mp) of 193 °C and retention factor (R_f_) of 0.23 (silica gel, CHCl_3_:MeOH 9.5:0.5). It is an ocher yellow spot when observed in UV light at λ = 366 nm, and when reacted with NH_3_ vapors, it is brown.

The ^1^H NMR spectrum of Chalcotanina showed nine signals integrating for 13 hydrogens, highlighting the presence of vinyl hydrogens (two signals), methoxy group hydrogens (two signals), and meta type coupling signals in ring A, indicating a tetra-substitution. Ring B has proton disposition in ortho arrangement, and a signal in meta position, indicating tri-substitution. The ^13^C NMR APT spectrum showed 17 signals, 3 signals for quaternary carbons, 7 signals for C-H carbons, 2 signals for O-CH_3_ carbons, and 5 signals for C-O carbons (Table 2) (Supplementary Figure S1). In the UV-Vis displacement analysis of Chalcotanina, due to presence of the AcONa + H_3_BO_3_ band, a hydroxyl substitution was evidenced in the C-3 and C-4 positions of B ring (ortho di-OH). A hydroxyl substituent in the C-4′ position is discarded, but the substitution of any other type of radical is possible, and it has a hydroxyl group in the C-4 position. The compound does not hydrolyze in the presence of acid (Table 3).

The exact calculated mass for the chalcone corresponded to 316.09299 ± 1.7 ppm obtained from the ESI negative ion mode, [M-H]- of 315.08808 and positive ion mode [M + H]+ of 317.09790, which suggested the molecular formula to be C_17_H_16_O_6_ (calc. 316.09469) (Supplementary Figure S2).

**Figure 2:**
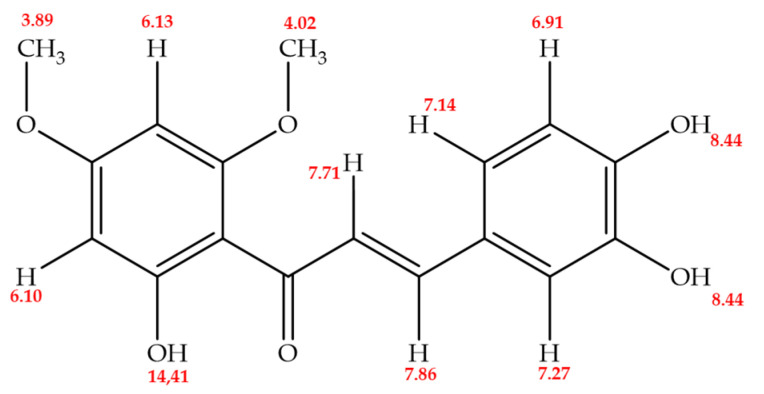
Elucidated structure of 2′,3,4-trihydroxy-4′,6′-dimethoxychalcone (Chalcotatina).

According to NMR and the UV spectral data, the compound was identified as 2′,3,4-trihydroxy-4′,6′-dimethoxychalcone (Figure 2), here called as Chalcotanina.

### 2.2. Anti-Cancer Potential of Chalcotanina

#### 2.2.1. Selective Cytotoxicity of Chalcotanina on Cancer Cells

**Figure 3:**
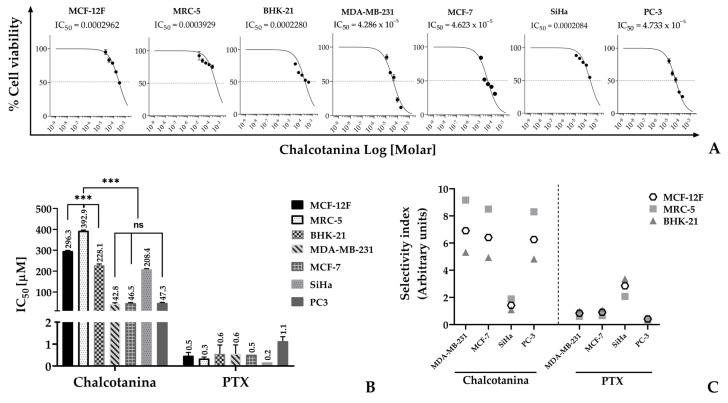
Cytotoxicity and selectivity of Chalcotanina (Cht) on cancer cells. (**A**) Percentage of cell viability, (**B**) half-maximal μM concentration (IC_50_), and (**C**) selectivity index of cancer cells. Data from three independent experiments, each performed in triplicate were carried out and data were analyzed in GraphPad Prism 8.0 (La Jolla, CA, USA). *p*-values indicate statistical significance (*** *p* < 0.001; ns: not significant).

The effect of Chalcotanina on cell viability was assessed using the MTT assay in four cancer cell lines: MDA-MB-231 and MCF-7 for breast cancer, SiHa for cervical cancer, and PC-3 for prostate cancer. Additionally, three normal cell lines were included, i.e., MCF-12F non-tumor mammary cells, MCR-5 lung fibroblasts, and BHK-21 kidney fibroblasts. The results indicate that cell viability decreases with increasing concentration of chalcone (Figure 3A). However, there is a significant difference in the calculated IC_50_ values, which determine the inhibition of cell proliferation, between normal and cancer cells (Figure 3B).

TNBC cells exhibited the highest sensitivity to Chalcotanina, with an IC_50_ value of 42.8 µM. The IC_50_ values against MCF-7 and PC-3 cells were not significantly different. Among the cancer cells, cervical SiHa cancer cells showed the highest resistance to treatment. The IC_50_ values for the normal cells MCF-12F, MRC-5, and BHK-21 were 296.3, 392.9, and 228.1 µM, respectively (Figure 3B). The selectivity index values indicated a strong selectivity of Chalcotanina for the TNBC cell line, with values of 6.9, 9.1, and 5.3 when compared to MCF-12F, MRC-5, and BHK-21 cells, respectively. In contrast, both the normal and cancer cells demonstrated high sensitivity to paclitaxel (PTX), resulting in selectivity index (SI) values of less than 4.0 (Figure 3C).

#### 2.2.2. Profiles of Annexin-V/7-AAD and Casp3/7 Activation in Chalcotanina-Induced Apoptosis in Cancer Cells

The induction of early and late apoptosis in the cells was evaluated in response to Chalcotanina using flow cytometric assays. PTX was used as a positive control. The Annexin-V assay and active-caspases 3/7 assays were conducted, both without and with the DNA dye 7-AAD, in order to distinguish between early and late apoptosis.

**Figure 4:**
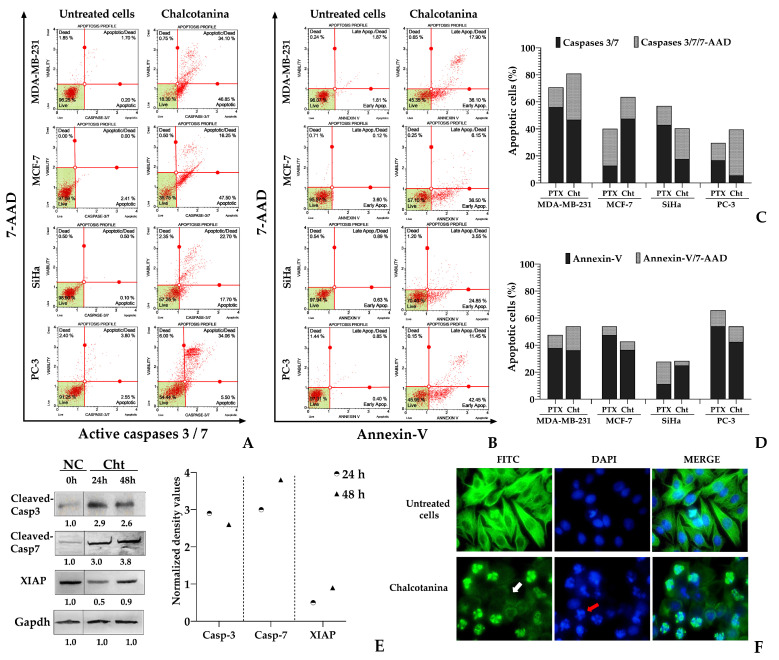
Early and late apoptosis induction on cancer cells exposed to Chalcotanina (Cht) at IC_50_. The dot (in red) represents a single analyzed cell, and in the green area, live cells are localized. (**A**) Activation of caspases 3/7. (**B**) Annexin V/7-AAD detection on cells. (**C**) The bar graph indicates the percentage of cells in early (Casp3/7-stained cells) or late (Casp3/7 and 7-AAD detection) apoptosis. (**D**) The bar graph indicates the percentage of cells in early (Annexin-V) or late (Annexin-V/7-AAD) apoptosis. NC corresponds to negative control cells, and PTX was used as the positive control. (**E**) Western blot analysis to evaluate the expression levels of effector caspases 3 and 7, as well as the anti-apoptotic XIAP. Protein expression was normalized to the negative control using density values obtained from Image J software. GAPDH was used as a loading control. (**F**) Immunofluorescence microscopy images showing the effects on microtubules (in green) and nuclei (in blue) in MDA-MB-231 cells exposed to Cht at 48 h. Images showed microtubule damage (white arrow) and nuclear condensation with the formation of apoptotic bodies (red arrow).

At 24 h, the results demonstrate the induction of apoptosis-mediated cell death in Chalcotanina-exposed cells. The activation percentages of caspases 3/7 ranged from 48% to 80%, and Annexin-V detection ranged from 28% to 54%, depending on the cell type (Figure 4A–F). According to the flow cytometric assays, early apoptosis was found to be the predominant status in breast cancer cells. However, a high percentage of SiHa and PC-3 cells exhibited both caspases 3/7 activation and the detection of the DNA dye reagent 7-AAD, indicating late apoptosis (Figure 4C,D).

Western blot independently confirmed the activation of cleaved caspases 3 and 7 during apoptosis in TNBC cells in response to Chalcotanina after 24 and 48 h. The results indicate a significant increase in the activation of both active caspases 3 and 7. However, it was observed that cleaved caspase-7 remained highly active at 48 h, even more so than caspase-3 (Figure 4E). Additionally, the expression of the caspase inhibitor protein XIAP was evaluated. It was found that XIAP decreased at 24 h due to the effect of Chalcotanina, but its levels increased again at 48 h (Figure 4E).

In addition, morphological analysis revealed damage to the integrity of microtubules and nuclei, characterized by chromatin condensation and the formation of apoptotic bodies. These observations provide visual evidence of apoptosis induction by Cht of TNBC cells (Figure 4F).

#### 2.2.3. Chalcotanina Induces Intrinsic Apoptosis via Depolarization of Mitochondrial Membrane Potential in TNBC Cells

**Figure 5:**
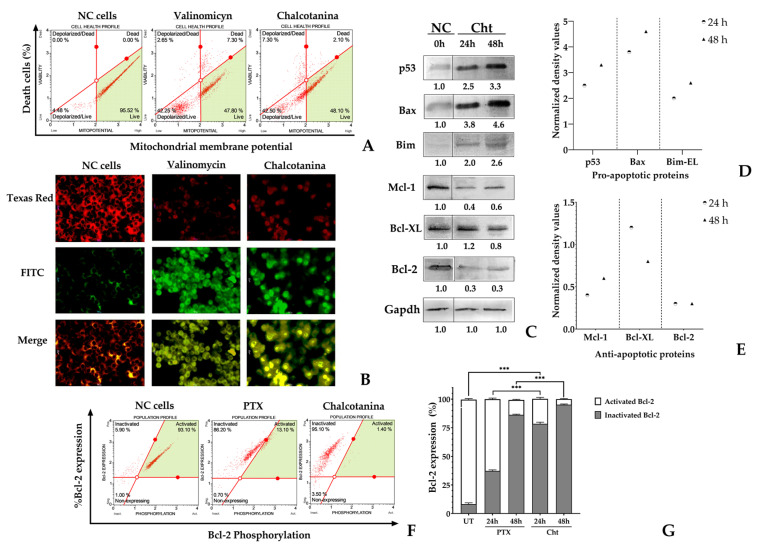
Mitochondrial apoptosis induction in TNBC cells in response to Chalcotanina (Cht) at IC_50_ was evaluated. (**A**) Flow cytometry was used for quantitative analysis of mitochondrial membrane potential. Each red dot represents an individual analyzed cell, and the green area represents the region of cells with polarized mitochondrial membrane. (**B**) Cells stained with the JC-10 dye were analyzed for fluorescence. Valinomycin at 100 nM served as a positive control, while untreated cells were used as the negative control (NC). (**C**–**E**) Western blot analysis was performed to assess the expression levels of p53, Bax, and Bim pro-apoptotic proteins, as well as anti-apoptotic proteins Mcl-1, Bcl-XL, and Bcl-2 in mitochondrial proteins. Protein expression was normalized to the negative control using density values obtained from Image J software. GAPDH was used as a loading control. (**F**,**G**) Flow cytometry was used to detect active Bcl-2-Ser70 in TNBC cells. Each red dot represents an individual analyzed cell, and the green area corresponds to cells with active Bcl-2. The bar graph indicates the percentage of active and inactive Bcl-2 protein expression in untreated and treated cells. Statistical significance is represented by *p*-values (*** *p* < 0.001).

Changes in mitochondrial inner membrane potential (ΔΨm) were assessed using both quantitative and qualitative fluorescent methods (Figure 5A,B). Additionally, a flow cytometry assay was performed, incorporating the DNA dye 7-AAD, to identify dead cells that exhibited ΔΨm loss upon exposure to Chalcotanina at IC_50_ for 24 h. The results revealed that approximately 42.5% of the cells exposed to Chalcotanina exhibited depolarization of the inner mitochondrial membrane (Figure 5A).

Based on the results of Western blot analysis and normalized densitometry data, it was observed that treatment with Chalcotanina led to an increase in the expression of pro-apoptotic proteins, including p53, Bax, and Bim. Conversely, the levels of two anti-apoptotic proteins, Bcl-2 and Mcl-1, were found to decrease. However, the expression level of the Bcl-2-like protein-1 (Bcl-XL) remained unchanged (Figure 5C–E).

The status of the Bcl-2 protein was assessed using flow cytometry to distinguish between its phosphorylated active form and its non-phosphorylated or inactive form. The study revealed that untreated cells predominantly displayed Bcl-2 in its active form. However, treatment with Chalcotanina resulted in a significant increase (approximately 95%) in its dephosphorylation compared to PTX (Figure 5F,G).

#### 2.2.4. Chalcotanina Targeting Autophagy in Cancer Cells

To detect autophagy, we monitored the lipidated LC3 protein (LC3B) present within the autophagosomes of cancer cells exposed to Chalcotanina for 48 h using flow cytometry. We further confirmed the results at 24 and 48 h through Western blot analysis of LC3B protein, the autophagy flux marker p62, and the negative regulator phospho-mTOR (Ser2448).

**Figure 6:**
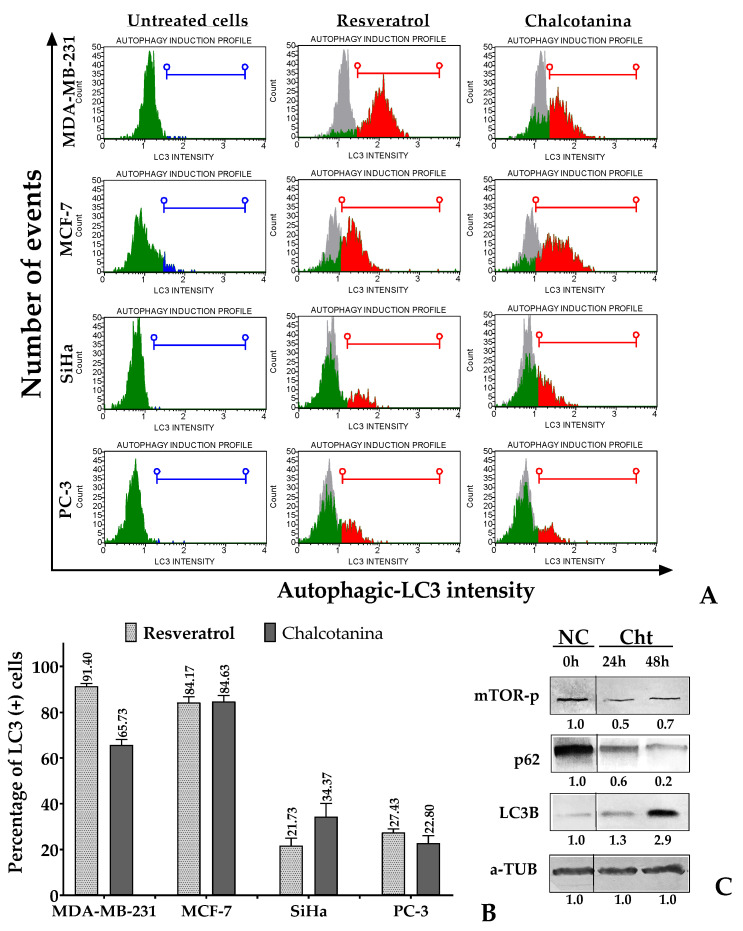
Analysis of autophagy induction in cancer cells in response to Chalcotanina (Cht). (**A**) Histogram plots from flow cytometry analysis representing the presence of autophagic LC3B protein inside cells. Cell population adjustments were made by using untreated cells as the negative control (NC) to determine the green population outside the blue line. Subsequently, we confirmed the population of autophagic cells by employing resveratrol-treated cells as the positive control, defined by the red line. (**B**) Percentage of cancer cells expressing LC3B+. (**C**) Western blot analysis of autophagic LC3B, p62, and mTOR-Ser2448 proteins. Semiquantitative analysis was performed by calculating the relative densitometric units using ImageJ and normalizing them against the negative controls.

The results of this study indicate a significant increase in LC3B protein levels in breast cancer cell lines when exposed to Chalcotanina. This increase was observed to a lesser extent in PC-3 prostate cancer cells (Figure 6A,B). Additionally, Western blot analysis confirmed a significantly increased expression of LC3B protein in MDA-MB-231 cells, accompanied by a decrease in p62 and mTORC1 phosphorylated proteins (Figure 6C).

### 2.3. Docking and Molecular Dynamics Demonstrate the Negative Modulation of Pro-Survival Proteins

In silico analyses were used to investigate the potential interactions between Chalcotanina (the ligand), and a set of five pro-survival proteins Bcl-2, Bcl-XL, Mcl-1, mTOR, and XIAP [77,78].

First, a molecular docking study was performed to elucidate the preferred binding modes and affinity of Chalcotanina to each protein. Subsequent MD simulations provided insights into the stability and dynamics of these protein–ligand complexes over time. To obtain a more accurate estimate of the binding free energy, the MM-PBSA (Molecular Mechanics Poisson–Boltzmann Surface Area) method was applied.

**Figure 7:**
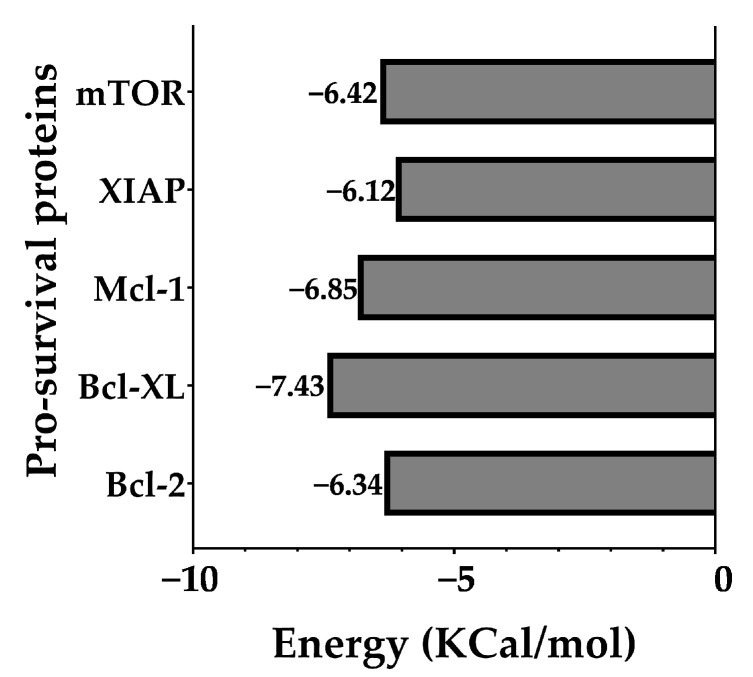
Docking results depict the binding affinities of the Chalcotanina ligand with each pro-survival protein, performed by Autodock Vina [79,80].

Molecular docking studies were executed using Autodock Vina [79,80]. The results, as illustrated in Figure 7, indicated a comparable binding affinity of the Chalcotanina ligand across the proteins. Notably, the Bcl-XL protein demonstrated marginally superior binding interaction. To further corroborate these findings and assess the stability of these protein–ligand complexes, subsequent molecular dynamics simulations were conducted.

The molecular dynamics (MD) simulations were initialized using the conformations derived from docking studies as starting structures. Each simulation was executed over a time span of 50 ns for individual protein–ligand complexes. The analysis of the root-mean-square deviation (RMSD) values, (Figure S3), revealed that the anti-apoptotic proteins maintained a stable interaction with the Chalcotanina ligand throughout the simulation time. A notable exception was observed for the negative regulator of the autophagy mTOR protein, which exhibited a significant conformational shift at approximately 15 ns.

The root-mean-square fluctuation (RMSF) (Figure S3) corroborates RMSD results, wherein mTOR demonstrates heightened fluctuations in comparison to the other proteins.

**Figure 8:**
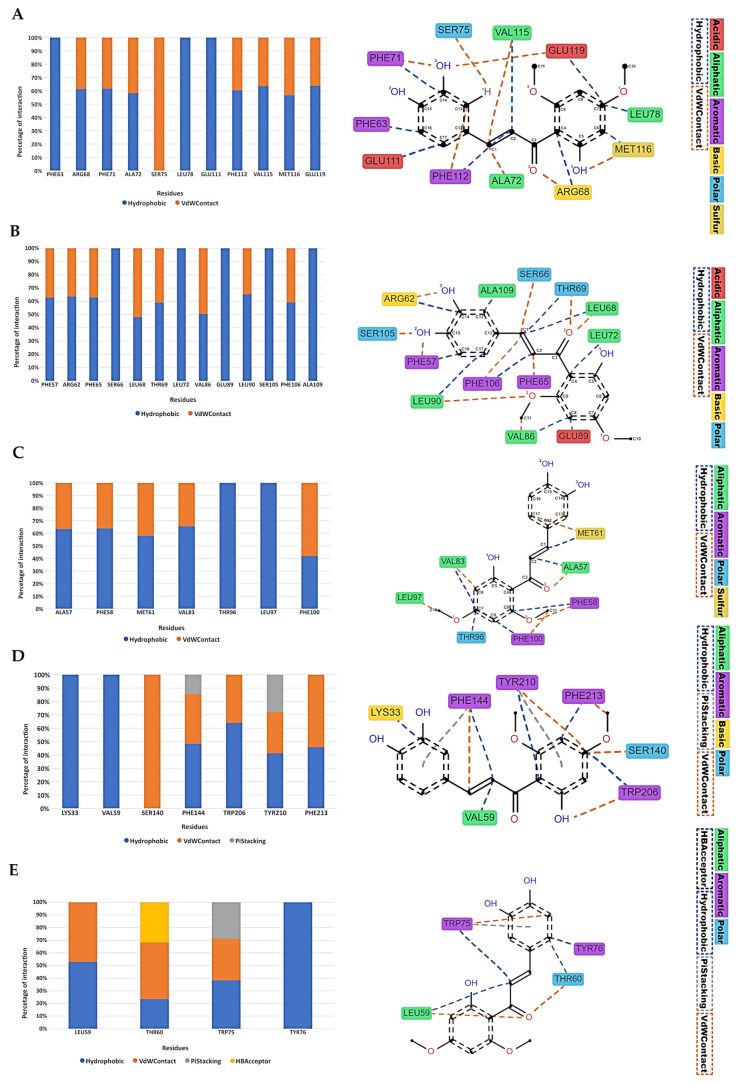
Nature of interactions between the residues of (**A**) Bcl-2, (**B**) Bcl-XL, (**C**) Mcl-1, (**D**) mTOR, and (**E**) XIAP proteins and the Chalcotanina (ligand at right) highlighting residues interacting for over 30% of the simulation duration.

To thoroughly examine the nature of the interactions throughout the molecular dynamic simulations, we used the Python library Prolif [81]. The entire simulation trajectory, encompassing all frames, was subjected to this analysis. The resultant interactions, specifically those persisting for more than 30% of the trajectory, are graphically depicted in Figure 8.

**Figure 9:**
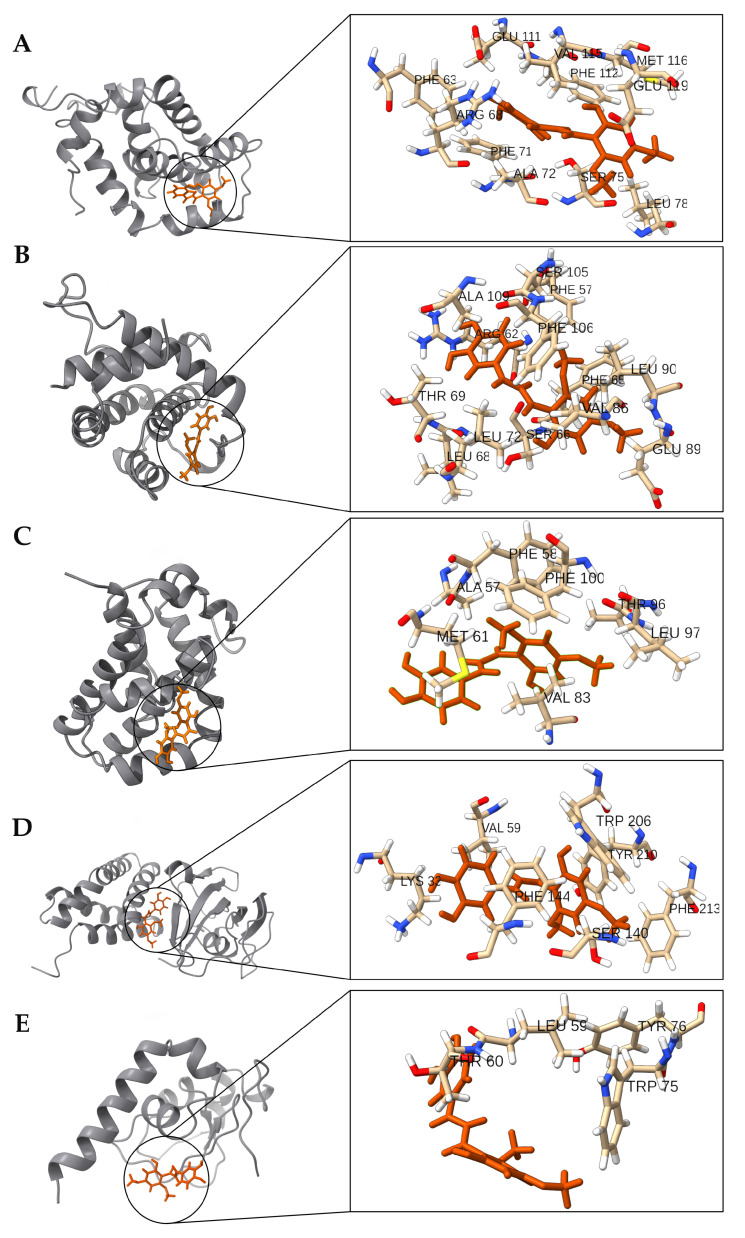
Comprehensive view of (**A**) Bcl-2, (**B**) Bcl-XL, (**C**) Mcl-1, (**D**) mTOR, and (**E**) XIAP proteins in complex with the ligand Chalcotanina; on the right, the main interacting residues are shown in detail. Proteins are shown in grey and Chalcotanina in orange.

In the interaction analysis of the anti-apoptotic proteins of the intrinsic pathway, two predominant types of interactions were observed with the ligand Chalcotanina: hydrophobic and Van Der Waals contacts (VdWContact) (Figure 8A–C and Figure 9A–C). For the mTOR protein, apart from the hydrophobic and VdWContact interactions, a notable π-Stacking interaction was identified with residues PHE144 and TYR210. On the other hand, XIAP protein manifested π-Stacking with TRP75 and HBAcceptor interactions with residue THR60 (Figure 8D,E and Figure 9D,E).

To discern the thermodynamics underpinning the stability of protein–ligand interactions, binding free energy calculations were executed utilizing the MM-PBSA method. This approach is esteemed for its balance between computational efficiency and accuracy, allowing for the extrapolation of free energy values from molecular dynamics trajectories [82].

**Figure 10:**
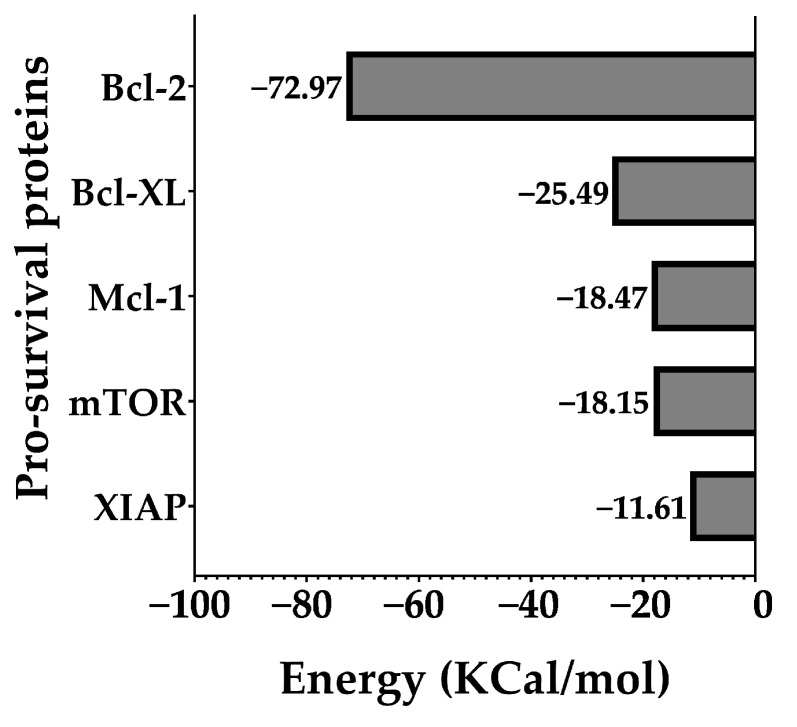
Binding free energy values, as calculated using MM-PBSA, for each pro-survival protein complexed with the ligand Chalcotanina.

For the MM-PBSA calculations, the final 2 ns of each trajectory were harnessed, ensuring the analysis was based on the most equilibrated phase of the simulation. According to the results (Figure 10), distinct variances in binding affinities across the proteins were observed. The most negative free energy released (−72.9) was evidenced for Bcl-2-ligand complex formation, while XIAP-ligand complex results in −11.6 free energy.

## 3. Discussion

Approximately ten chalcones from the genus Chromolaena have been isolated and identified, with nine of them described by Ojo et al. in 2022 [14]. In the present study, a novel chalcone named Chalcotanina was successfully isolated and identified from the inflorescences of *Ch. tacotana*. It has a calculated molecular weight of 316.09299 amu (C_17_H_16_O_6_).

The ^1^H NMR spectrum exhibits characteristic signals for methoxyl groups in higher chemical shift regions, indicating peaks at δ = 3.89 and 4.82 ppm. Furthermore, signals around δ = 7.71 and 7.91 ppm are observed, with each integrating for one hydrogen, corresponding to vinyl hydrogen atoms coupled to α and β carbons, a typical feature of chalcones. At δ = 6.10 and 6.13 ppm, a coupling constant (JHH) of 2.4, suggestive of meta-coupling on ring A, is evident, suggesting a meta-tetra-substituted system. Regarding ring B, signals at δ = 6.10 and 6.13 ppm confirm the ortho-position relationship between the respective protons. The signals at 7.14 ppm (dd, *J* = 8.3, 1.9 Hz) and 7.27 ppm (d, *J* = 2.1 Hz) on ring B are coupled in the meta-position, indicating that ring B is tri-substituted (Table 1). The ^13^C NMR APT spectrum exhibits 17 signals, each corresponding to a carbon atom. At δ = 192.42 ppm, the carbonyl carbon signal, typical of a flavonoid structure, is clearly observable. The signals at δ = 55.61 ppm and 55.17 ppm in the negative phase can be attributed to methoxyl groups. Chalcotanina presents six distinctive carbon signals in the negative phase that are coupled to protons (CH), while the remaining signals correspond to quaternary carbons. Notably, the signals at 168.26, 166.36, 162.77, 148.11, 145.48, and 145.54 ppm are associated with the presence of an oxygen atom. The COSY H-H spectrum reveals the vicinal coupling between the C-α and C-β protons, confirming the presence of vinylic protons in the structure and the ortho-position of two methines (CH) in ring B.

Based on the connectivity of the various hydrogens with their corresponding carbons, as determined from HMQC and HMBC experiments, the compound under investigation is identified as 2′,3,4-trihydroxy-4′,6′-dimethoxychalcone (Figure 1). The NMR data for this chalcone exhibit similar assignments to those reported for a newly synthesized chalcone, with fifteen peaks evident in the ^13^C NMR spectrum. The peak at 193.2 ppm is attributed to a carbonyl group. Notably, three peaks displaying doubled intensities at 55.6, 106.5, and 161.2 ppm are assigned to 3′-OMe/5′-OMe, C-2′/C-6′, and C-3′/C-5′, respectively. Additionally, the peak at 55.9 ppm is designated for 2-OMe′ [83].

The experimentally calculated mass agrees with the structure elucidated using NMR, with variations of less than 5 ppm. Chalcones were readily deprotonated due to their acidic nature. The loss of *m*/*z* 30 was attributed to the sequential loss of CH3 (*m*/*z* 15). This effect can be observed in the fragment at *m*/*z* 285. Fragment ions at *m*/*z* 153 and 109 correspond to cleavages resulting from losses in A and B rings, confirming the location of the methoxyl groups in ring A. Similar results were reported by Zhang, who characterized the structures and commonly observed losses including H_2_, H_2_O, CO, CO_2_, and methyl radicals in chalcones containing methoxy groups. It was also indicated that specific neutral losses occurred through stepwise pathways, such as the neutral loss of *m*/*z* 44 as CH_3_ and HCO, or CH_4_ and CO, in addition to CO_2_ [84].

There have been numerous studies conducted on the cytotoxic activity of natural or synthetic chalcones. However, it is unfortunate that most of these studies do not provide information on the selectivity index (SI). The SI is defined as the ratio of the toxic concentration to the effective bioactive concentration in normal cells [85]. In our study, we investigated the cytotoxicity of Chalcotanina, a specific chalcone, on breast and prostate cancer cells, as shown in Figure 3A,B. Interestingly, we found that Chalcotanina exhibited cytotoxic effects on these cancer cells but not on cervical cancer cells. Moreover, we evaluated the cytotoxicity on non-tumor cells to calculate the SI, and the results revealed SI values above 4.0 for the cancer cells, except for SiHa cells (Figure 3C). These results indicate that Chalcotanina may cause less damage to healthy neighboring tumor cells. According to Weerapreeyakul et al. [86], SI values above 3.0 are considered indicative of a potential anti-cancer agent. This information is crucial for determining whether further studies should be pursued.

A few studies have reported a correlation between the antiproliferative activity of natural chalcones and the induction of apoptosis. These studies have investigated chalcones such as butein, flavokavains A, B, and C, the prenylated chalcone xanthohumol, and isoliquiritigenin. Notably, isoliquiritigenin has also been found to induce autophagy in breast cancer cells [87].

Apoptosis is characterized by a series of molecular events and morphological changes that allow for the distinction between early and late stages of the process. One of the early events is the activation of the molecular machinery that subsequently induces the characteristic morphological changes in apoptotic cells [88]. In this context, the activation of effector caspases 3 and 7 is crucial for ensuring apoptosis activation. In a previous study, it was determined that caspase-3 is essential for chromatin condensation and DNA fragmentation, while caspase-7 is involved in the production of reactive oxygen species (ROS) and the detachment of apoptotic cells from the extracellular matrix [89]. In this study, we demonstrated that chalcotanin induces the formation of apoptotic bodies and affects the integrity of microtubular dynamics 48 h after treatment (Figure 4F). This effect may be associated with the activation of caspase-3 and caspase-7, as we observed a rapid and significant increase in the activation of effector caspases 3/7, particularly caspase-7, with chalcotanin treatment (Figure 4E). Additionally, we observed phosphatidylserine (PS) exposure in approximately 40% of the cells under the same conditions. This result suggests that the activation of caspases 3/7 occurs before the translocation of PS to the extracellular environment. Furthermore, our findings support a previous study which proposed that changes in the outer membrane of apoptotic cells, such as the externalization of PS, depend on the activation of caspase-6 and caspase-7 rather than caspase-3 [90].

However, the activation of caspases requires the inactivation of XIAP, which is a protein known to be the major member of the IAP family. XIAP directly inhibits caspases 3 and 7 by blocking the caspase active site through the formation of a complex with the BIR2 domain in the N-terminal of XIAP [91]. Our results suggest that chalcotanin may negatively modulate XIAP, potentially facilitating the uncoupling between XIAP and caspases 3 and 7. This is supported by data from docking and molecular dynamics simulations (Figure 7, Figure 8, Figure 9 and Figure 10) despite the low impact on protein amounts observed after treatment (Figure 4E).

Previous studies have demonstrated that hydroxychalcones, including butein and isoliquiritigenin (ISL), can reduce the expression of Bcl-2 and induce caspase-3 cleavage. This caspase-3 cleavage has been associated with the cleavage of poly(ADP-ribose) polymerase (PARP) [92,93].

Our results suggest that chalcotanin triggers the intrinsic pathway of apoptosis. We observed elevated levels of pro-apoptotic proteins p53, Bax, and Bim (Figure 5C,D), and decreased levels of pro-survival proteins Mcl-1 and Bcl-2. It is worth noting that there was no significant reduction in Bcl-XL protein levels in TNBC cells (Figure 5C,E).

Bcl-2 is a well-known anti-apoptotic protein that could inhibit Bax or Bak proteins. It can also inhibit the activities of caspase-3 and -7 [94]. In addition, Bcl-XL is a protein that is often overexpressed in breast cancer and contributes to metastasis [95]. However, our findings suggest that the induction of apoptosis is independent of the levels of Bcl-XL. Instead, it relies on the reduction of Bcl-2 and Mcl-1 proteins. Similar to Bcl-2, Mcl-1 acts by sequestering Bak or Bax, but it has a high affinity for Bim protein [96]. Bim is a pro-apoptotic protein that is increased in response to chalcotanina.

In addition, Chalcotanina caused the dephosphorylation of Bcl-2 at serine 70 in a significant proportion of cells (Figure 5F,G). It has been reported that phosphorylated Bcl-2 is necessary for the formation of anti-apoptotic heterodimers with Bax or Bak, which in turn leads to the preservation of permeability in the outer mitochondrial membrane and the inhibition of apoptosis [97]. However, our findings showed that exposure to Chalcotanina resulted in the depolarization of the mitochondrial membrane potential in a high percentage of MDA-MB-231 cells (Figure 5B), as well as a significant increase in Bax, which promotes intrinsic apoptosis induction (Figure 5C,D).

Furthermore, we demonstrated that Chalcotanina can induce apoptosis and autophagy in a time-dependent manner, as evidenced by the high levels of LC3B protein, as well as the inactivation of p62 and mTOR (Figure 6). Only a few studies have explored the ability of natural hydroxy-methoxy-chalcones to simultaneously induce apoptosis and autophagy. One such compound, FKB, has been shown to induce autophagy in melanoma cells by causing the accumulation of microtubule-associated protein 1A/1B and light chain 3B (LC3B), downregulating the expressions of protein kinase B (AKT)/mammalian target of rapamycin (mTOR), and disrupting Bcl-1/Bcl-2 levels [98]. Additionally, it has been reported that targeted siRNA-mediated silencing of Bcl-2, a common regulator of apoptosis and autophagy, can induce autophagic cell death in breast cancer cells overexpressing Bcl-2.

The experimental results were supported by in silico analysis, where the anti-apoptotic proteins Bcl-2, Mcl-1, and XIAP exhibited minimal variations in their RMSD trajectories (Figure S3A–E). This suggests that Chalcotanina does not induce pronounced conformational changes. This observation is further supported by the RMSF plots (Figure S3F–J). Elevated fluctuations at the termini of these proteins can be ascribed to their terminal loop regions, which are inherently flexible and exhibit high mobility (Figure 9). Conversely, a crucial observation arises from the binding mode of Chalcotanina on mTOR (Figure 8D and Figure 9D).

As depicted in Figure 9, the Chalcotanina interacts at the interface of a dimeric mTOR structure. From a biophysical standpoint, the ligand’s location at the dimer interface can have profound implications. Dimer interfaces are critical regions for protein–protein interactions and play a pivotal role in maintaining the structural integrity and functional capacity of the dimeric protein complex. The insertion or binding of a ligand at such an interface can lead to steric hindrance, disrupting the delicate balance of interactions that hold the two monomers together. This can result in altered protein dynamics, reduced stability, and potentially modified activity [99]. The introduction of Chalcotanina at the mTOR dimer interface may not only perturb its inherent stability but also impede the protein’s native function, thereby presenting a potential mechanism of action for the ligand. Differences in the type of interactions of protein ligand were found, while Bcl-2 family member proteins include hydrophobic and VdWContact, and XIAP and mTOR also present a π-Stacking interaction (Figure 8). It is well established in the literature that hydrophobic interactions play a pivotal role in stabilizing protein–ligand complexes [100,101]. This arises from the entropic benefit achieved when water molecules that initially surround the hydrophobic moieties are released upon ligand binding, leading to an increase in the system’s entropy and subsequently stabilizing the complex. Moreover, these interactions minimize the exposure of hydrophobic regions to the aqueous environment, further stabilizing the protein–ligand conformation. According to the results of MM-PBSA calculations (Figure 10), and the binding free energy values, one can infer the probable molecular interactions contributing to such energies. The considerably favorable energy value for Bcl-2 suggests an intricate network of interactions including hydrogen bonding, hydrophobic contacts, and potential ionic interactions that collectively stabilize the Chalcotanina ligand within the protein’s binding pocket. Additionally, the solvation effects, evaluated by the Poisson–Boltzmann Surface Area component of MM-PBSA, could play a role in this stabilization, emphasizing the importance of solvent in mediating these interactions [102]. On the contrary, proteins like XIAP and mTOR, although manifesting favorable interactions, do not reach the magnitude of binding energy exhibited by Bcl-2. This could be attributed to either lesser optimal interactions or potential repulsive forces, underscoring the intricate balance of forces in protein–ligand interactions. Furthermore, factors such as ligand strain upon binding and entropic contributions could also impact the observed binding energies. It is pivotal to understand that while binding free energy offers vital insights into the stability of the complex, it is a cumulative measure of various energetic components, each bearing its significance in the binding process.

## 4. Materials and Methods

### 4.1. Chalcone Extraction and Isolation

The plant material was collected in La Vega, Cundinamarca (Colombia), at an elevation of 1250 m above sea level (m.a.s.l.). A specimen plant was deposited at the Herbarium of Pontificia Universidad Javeriana, Bogotá, Colombia, and was identified as *Chromolaena tacotana (Klatt)* R. M. King and H. Rob, and assigned voucher number HPUJ30170.

The dried inflorescences were ground in a mill (RTE-648, Tecnal, Sao Paulo, Brazil) and sifted through a 60-mesh sieve (0.25 mm) (EMS-08, Electrolab, Mumbai, India) after being dried at 60 °C with <8% humidity (MKF, Binder, Tuttlingen, Germany). A total of 490.17 g of powder was subjected to Soxhlet extraction using petroleum ether (Sigma-Aldrich, St Louis, MO, USA). Subsequently, a dichloromethane (CH_2_Cl_2_) (Merck Group, DE, Darmstadt, Germany) extraction was performed to remove fats and chlorophylls from the content. The solvent was evaporated using a rotary vacuum evaporator at 60 rpm (R-300, BÜCHI Labortechnik AG, Flawil, Switzerland) at 45 °C, and the resulting material was weighed (DCI fraction: 30.82 g) (Explorer, Ohaus, NJ, USA).

The DCI fraction was flocculated with methanol (MeOH)/water (1:1) (Merck Group, DE, Darmstadt, Germany), and liquid–liquid extraction with CH_2_Cl_2_ was performed to obtain the fraction of interest (DC-II). A total of 7.8563 g of the DC-II fraction was separated with column chromatography (CC) using silica gel 63–200 μm (Silicagel 60, Merck Group, DE, Darmstadt, Germany) and eluted with petrol/ethyl acetate (AcOEt) (Merck Group, DE, Darmstadt, Germany) in ratios of 8:2, 7:3, and 6:4, followed by ethanol (EtOH) (Merck Group, DE, Darmstadt, Germany). This process resulted in the isolation of 79 µg of the Chalcotanina compound, which was subsequently purified and crystallized through successive washing with acetone (Merck Group, DE, Darmstadt, Germany).

### 4.2. Structural Identification

^1^H and ^13^C NMR data were obtained using the Avance Bruker 300 spectrophotometer (Bruker Biospin, Mannheim, Germany) operating at a frequency of 300 MHz for hydrogen and 75 MHz for carbon, respectively, and the spectra were measured in acetone-d6 (Merck Group, DE, Darmstadt, Germany). Mass data were obtained in an ultra-high-pressure liquid chromatography (UPLC) coupled with a quadrupole time-of-flight mass spectrometer detector (QTOF), Shimadzu (LCMS-9030) (Kioto, Japan) by direct injection. O-CH_3_ and OH positions were obtained using UV-VIS 230 to 500 nm scanning (Jenway 6405 UV-VIS, Staffordshire, UK) with displacement reagents (AlCl_3_, HCl, AcONa, MeONa, and H_3_BO_3_) (Merck Group, DE, Darmstadt, Germany).

### 4.3. Cell Lines, Culture, and Analysis

The human TNBC MDA-MB-231 (HTB-26) cell line, the luminal A subtype expressing hormone receptors MCF-7 adenocarcinoma cells, and the cervical adenocarcinoma SiHa cells were grown in RPMI 1640 (Lonza, Basel, Switzerland) medium. The androgen-independent prostate PC-3 cell line was grown in EMEM (Lonza, Basel, Switzerland). All media for cancer cells were supplemented with 10% heat-inactivated fetal bovine serum (Biowest, Nuaillé, France, Bradenton, FL, USA) and 1.0% penicillin/streptomycin (Lonza, Basel, Switzerland). The normal fibroblast cells MRC5 (CCL-171) and the BHK-21 kidney fibroblasts were grown in supplemented EMEM. The normal epithelial MCF-12F (CRL-10783) mammary gland cells were grown in DMEM/F-12 (Sigma-Aldrich, St Louis, MO, USA) supplemented with 7.0% fetal horse serum (Sigma-Aldrich), 10 µg/mL human insulin (Sigma-Aldrich, St Louis, MO, USA), 20 ng/mL epidermal growth factor (Sigma-Aldrich), 500 ng/mL hydrocortisone (Sigma-Aldrich, St Louis, MO, USA), 100 mg/mL cholera toxin (Sigma-Aldrich), and 1.0% penicillin/streptomycin (Lonza, Basel, Switzerland). All cell lines were obtained from the American Type Culture Collection (ATCC, Manassas, VA, USA).

### 4.4. Cell Viability Assay and Selectivity of Chalcones

Effects on cell viability were determined by the 3-(4,5-methyl-thiazol-2-yl)-2, 5-diphenyl tetrazolium bromide (MTT) assay. Cells were seeded at a density of 8000 cells per well on 96-well plates and incubated overnight to allow cell attachment at 37 °C and 5.0% CO_2_ to allow cell attachment. Cells were treated with Chalcotanina at 48 h with a concentration ranging between 100 and 1.0 μg/mL.

Cells treated with 0.5% DMSO molecular grade (Thermo-Fisher Scientific, Waltham, MA, USA) were used as a negative control. After 48 h of incubation, 0.5 μg/mL MTT (Sigma-Aldrich, St Louis, MO, USA) was added and incubated for 3.5 h. The formazan product was solubilized in DMSO, and absorbance values were measured at 570 nm using a microplate reader (Bio-Rad, Hercules, CA, USA). The inhibitory concentration required to reduce cell viability to 50% (IC_50_) relative to untreated negative control cells was estimated with nonlinear regression using GraphPad Prism 8.0, (La Jolla, CA, USA). The selectivity index was calculated as the ratio of IC_50_ on normal cells/IC_50_ on cancer cells [85].

### 4.5. Detection of Apoptosis Induction in Response to Chalcotanina

#### 4.5.1. Flow Cytometry

Detection of live cells, early and/or late apoptosis was performed using both Muse^®^ Annexin V & Dead Cell Reagent Kit (Luminex Corporation, Austin, TX, USA) and Muse^®^ Caspase-3/7 Kit (Luminex Corporation, Austin, TX, USA). In each test, 50.000 MDA-MB-231 cells were seeded per well in a 24-well plate, and after attachment, they were treated with Chalcotanina at IC_50_ for 24 h, and Paclitaxel (Sigma-Aldrich, St Louis, MO, USA) was used as the positive control. Cells in 0.5% molecular grade DMSO (Thermo-Fisher Scientific, Waltham, MA, USA) served as the negative control. After treatments, both the medium and the harvested cells were centrifuged, washed in 1× PBS, resuspended, and stained with reagents solutions according to the manufacturer’s instructions in each respective Muse^®^ Kit. Apoptotic cells were detected on the Guava^®^ Muse^®^ Cell Analyzer (Luminex Corporation, Austin, TX, USA) and analyzed in the Muse^®^ software 1.8. (Luminex Corporation, Austin, TX, USA)

#### 4.5.2. Western Blot

Untreated MDA-MB-231 cells and cells treated with Chalcotanina for 24 and 48 h were collected, lysed with buffer (20 mM Tris HCl pH 8.0 (Thermo-Fisher Scientific, Waltham, MA, USA), 137 mM NaCl (Merck Group, DE, Darmstadt, Germany), 10% glycerol (Sigma-Aldrich, St Louis, MO, USA), 1.0% NP-40 Surfact-Amps™ (Thermo-Fisher Scientific, Waltham, MA, USA), and 10 mM EDTA (Sigma-Aldrich, St Louis, MO, USA), and the supernatant was collected to obtain the total protein extract. The bicinchoninic acid (BCA) assay (Pierce) was used to measure the concentration of the total protein. A total of 40 μg of protein was loaded in 10 or 12% SDS-PAGE. Proteins were blotted using polyvinylidene fluoride (PVDF) membranes (Thermo-Fisher Scientific, Waltham, MA, USA), subsequently blocked for 1 h with 5.0% (*w*/*v*) BSA/TTBS (Thermo-Fisher Scientific, Waltham, MA, USA) and incubated overnight at 4 °C with the primary antibodies anti-caspase-7 (GTX31704), anti-cleaved-casp-3 (Thermo-Fisher Scientific, Waltham, MA, USA), anti-XIAP (D2Z8W), and anti-GAPDH (GT239) (Genetex, Irvine, CA, USA), and the latter was used as the loading control. The membranes were incubated for 2 h at room temperature, with anti-mouse IgG or anti-rabbit IgG (1:5000) (Sigma-Aldrich, St Louis, MO, USA) [16]. Bands detection was performed using the chromogenic HRP system 1-Step™ TMB-Blotting Substrate Solution (Thermo-Fisher Scientific, Waltham, MA, USA), yielding a blue-colored precipitate. ImageJ paper free software was used to normalize and semi-quantify bands’ density.

#### 4.5.3. Morphological Analysis of Nuclei and Microtubules with Epifluorescent Microscopy

TNBC cells were cultured in a 24-well plate. After adherence and growth to 70% confluence, cells were treated with Chalcotanina and incubated at 37 °C in 5.0% CO_2_ for 24 h. DNA staining was performed with DAPI (Sigma-Aldrich, St Louis, MO, USA), and microtubules were labeled with anti-alpha tubulin antibody T9026 (Sigma-Aldrich, St Louis, MO, USA) and Alexa Fluor 388 (FITC) (Sigma-Aldrich, St Louis, MO, USA). Images were captured using a MoticCamPro 282A and analyzed at 40× using Motic Image Plus 2.0 software (Schertz, TX, USA)

### 4.6. Analysis of Intrinsic Pathway of Apoptosis in TNBC cells

#### 4.6.1. Changes in the Mitochondrial Membrane Potential (ΔΨm)

To analyze mitochondrial trans-membrane potential, (ΔΨm) in response to Chalcotanina, cytometric flow and immunofluorescent analyses were carried out. One hundred thousand cells were seeded in a 12-well plate, and after incubation over-night at 37 °C and 5.0%, CO_2_ adherent cells were exposed at IC_50_ of Chalcotanina and 100 nM Valinomycin (Sigma-Aldrich, St Louis, MO, USA) for 24 h. The Muse^®^ MitoPotential Kit (Luminex Corporation, Austin, TX, USA) was used to detect if the compound induces depolarization of the mitochondrial membrane and 7-AAD as an indicator of cell death. The ΔΨm in the TNBC cells were detected on the Guava^®^ Muse^®^ Cell Analyzer (Luminex Corporation, Austin, TX, USA) and analyzed in the Muse^®^ software 1.8. (Luminex Corporation, Austin, TX, USA)

For immunofluorescent analysis, about 50.000 cells were seeded in a 12-well plate, and after 24 h of Chalcotanine treatment, the changes in the mitochondrial membrane potential were analyzed using a solution of 20 µg/mL JC-10 probe (Ultra-pure-Enzo Life Science, Farmingdale, NY, USA) dissolved in DMSO (Sigma-Aldrich, St Louis, MO, USA). Images were captured after 30 min of incubation at 37 °C, using a Motic-CamPro 282A (Motic Deutschland GmbH. Wetzlar, Germany) and analyzed at 40× using Motic Image Plus 2.0 software (Motic Deutschland GmbH. Wetzlar, Germany).

#### 4.6.2. Pro- and Anti-Apoptotic Proteins Analysis by Western Blot

A 40 μg quantity of the total protein, obtained from untreated MDA-MB-231 cells and cells treated with Chalcotanina for 24 and 48 h, was processed as described in 4.5.2, using the flowing antibodies: anti-Bim-EL (GT1234), anti-Bcl-2 (N1N2) (GeneTex, Irvine, CA, USA), anti-Bax (D2E11), anti Bcl-XL (54H6), and anti-Mcl-1 (D2W9E) (Cell signaling Technology, Danvers, MA, USA) (to identify pro- and anti-apoptotic proteins levels).

#### 4.6.3. Phospho-Bcl-2 Protein Detection

The Bcl-2 protein phosphorylation at serine 70 (S70pBcl-2) was analyzed using flow cytometry. Two hundred thousand cells were seeded in a 12-well plate, and after incubation overnight at 37 °C and 5.0%, CO_2_ adherent cells were exposed to IC_50_ of Chalcotanina and PTX for 24 and 48 h. To measure the Bcl-2 protein, we follow the instructions suggested in the Muse™ Bcl-2 Activation Dual Detection Kit (Luminex Corporation, Austin, TX, USA) that includes two directly conjugated antibodies, anti-phospho-Bcl-2 (Ser70)-Alexa Fluor^®^555 and anti-Bcl-2-PECy5 to detect inactive Bcl-2 expression. Treated and untreated cells were dissociated and harvested for fixation, permeabilization, staining, data acquisition, and analysis using Muse™ Cell Analyzer software. Three replicates were analyzed under the same conditions. Results are presented as the means of the replicates.

### 4.7. Autophagy Induction in TNBC Cells Exposed to Chalcotanina

To investigate the autophagy induction, an analysis of the intracellular expression of LC3B protein was performed using flow cytometry with the Muse^®^ Autophagy LC3B-antibody Based Kit (Luminex Corporation, Austin, TX, USA), following the manufacturer’s instructions. Approximately 30,000 cells per well were seeded in a 48-well plate and incubated overnight at 37 °C and 5.0% CO_2_. Cells were treated with Chalcotanina and with resveratrol used as a positive control [103] at IC_50_. Untreated cells with 0.5% DMSO were left in supplemented growth medium. After 24 h of treatment, and a selective permeabilization with the reagent A, cells were harvested and washed with 1× PBS. The Anti-LC3B Alexa Fluor™ 555 was added to each sample tube, leaving on ice for 30 min in the dark. Later, the cells were resuspended within the buffer assay 1× from the kit. The data acquisition of autophagy protein LC3B was performed on the MUSE cell analyzer (Luminex Corporation, Austin, TX, USA). During acquisition, live statistical values are generated and represented on a histogram graph.

In addition, 40 μg of the total protein, obtained from untreated MDA-MB-231 cells and cells treated with Chalcotanina for 24 and 48 h, was processed as described in 4.5.2, using the flowing antibodies, i.e., anti- mTOR-Ser2448 (Proteintech), anti- LC3B, and anti-p62 (GeneTex, Irvine, CA, USA), to detect autophagic markers, and anti-α-Tubulin-T9026 (Sigma-Aldrich, St Louis, MO, USA) as loading control.

### 4.8. Statistical Analysis for Biological Test

Data were expressed as the mean of three biological replicates (*n* = 3) ± SD (standard deviation), and the difference between control and treatments were determined using a two-way ANOVA test and Tukey multiple comparison test. The statistical analysis test was performed using GraphPad Prism 8.0, (La Jolla, CA, USA). A *p*-value < 0.05 ** or <0.001 *** was considered a statistically significant difference between the means.

### 4.9. Molecular Docking and Molecular Dynamics

Using Autodock Vina version 1.2.0 and the Vina force field as stated in references [79,80], docking simulations were run. A cubic box with side lengths of 35, along each axis, was centered around the ligands to determine the search parameters. The area that will be sampled by the docking algorithm was determined by this cubic box.

The default value of 0.375 for the grid spacing, which designates the distance between points in the search grid, was chosen. This setting is important because it affects how detailed the molecular docking study will be. Given that this parameter influences how comprehensive the search method is, a higher exhaustiveness value of 50 was selected to increase the accuracy of the simulation results.

The docking protocol utilized the best docking pose as the initial conformation for subsequent molecular dynamics simulations. This approach enhances the accuracy of the computational studies, as the initial docking pose typically represents a low-energy and possibly near-native conformation.

For the protein structures, they were retrieved from the Protein Data Bank (PDB). Specifically, the PDB IDs for the selected proteins were as follows: Mcl-1 with PDB ID 5FDR as per reference [78], Bcl-XL with PDB ID 3ZLN, Bcl-2 with PDB ID 3ZLN, XIAP with PDB ID 5OQW, and mTOR with PDB ID 4JSV. In order to facilitate the docking simulations, protein structures co-crystallized with an inhibitor were given preference. In the event that such structures were unavailable, the Castp server was employed to identify and analyze cavities in the protein structures.

Using the ChimeraX program, all water molecules and other solvents were eliminated before docking. The ADRF suite was then used to convert the proteins to the pdbqt format, as explained in reference [80]. Since it contains both partial atomic charges and atomic coordinates, this format is essential for Autodock Vina.

The MEEKO software, which can be downloaded from https://github.com/forlilab/Meeko (accessed on 1 December 2021), was used to prepare the ligands. In the event where polar hydrogens are missing from the original structures, this software makes it easier to add them and computes Gasteiger partial charges. The docking simulations employed the resulting pdbqt files for the ligands, which contained both the spatial and electrostaticc properties of the molecules.

#### 4.9.1. Molecular Dynamics Simulation Preparations

For molecular dynamics, recalculating the charges allowed for a more thorough molecular description of the ligands. According to reference [104], the charges were specifically calculated using Gaussian 16 and the B3LYP/6-311g basis set. The Antechamber module was then used to create the coordinate and parameter files for the ligands.

Following that, Leap, a tool included in the AmberTools suite as defined in reference [105], was used to create the protein–ligand system. The General Amber Force Field (GAFF) for the ligand [106] and ff14SB for the protein [107] were selected as the force fields for this step.

Subsequent system solvation was executed with water molecules using the TIP3P water model [108]. To achieve electrical neutrality in the system, sodium (Na+) or chloride (Cl−) ions were included as necessary. This comprehensive system preparation methodology ensures an accurate starting point for subsequent computational analyses.

#### 4.9.2. Molecular Dynamics Configuration

Molecular dynamics simulations were performed using Amber18 [105]. The initial steps involved energy minimization of the water molecules. This was conducted using a maximum of 5000 cycles (maxcyc = 5000), transitioning from steepest descent to conjugate gradient optimization after 1000 cycles (ncyc = 1500). The SHAKE algorithm was not applied (ntf = 1), and the solute was constrained with a force constant of 100 kcal/mol/Å^2^.

Following this, energy minimization of the entire system was carried out. In this instance, the procedure was executed with a maximum of 100,000 cycles (maxcyc = 100,000), once again switching from steepest descent to conjugate gradient after 1000 cycles (ncyc = 1000), and the SHAKE algorithm was kept deactivated (ntf = 1).

Next, the system was gradually heated to a temperature of 300 K over a period of 500 picoseconds (ps), with the solute held fixed using a force constant of kcal/mol/Å^2^. This heating phase employed a canonical (NVT) ensemble. Subsequently, the system pressure was equilibrated over a span of 500 ps using an isothermal–isobaric (NPT) ensemble. Following these preparatory stages, the system was subjected to an equilibration phase of 1 nanosecond (ns).

During the production phase, each system was run for a total of 10 ns. The final 2 ns of these simulations were utilized for the MMPBSA calculations, consistent with methodologies utilized in previous work [109,110]. This comprehensive approach helps to ensure a robust exploration of the system’s phase space.

#### 4.9.3. MMPBSA

The binding free energy calculations were performed using the MMPBSA.py module integrated within the AmberTools18 suite [111]. For these analyses, 200 snapshots were extracted from the simulation trajectories. This quantity of snapshots represents the final 2 nanoseconds (ns) from the production phase of the molecular dynamic simulations, sampled at a frequency consistent with the parameters inp = 1 and radiopt = 0. For accurate calculation of binding free energies, this snapshot selection technique offers a sufficiently wide range of conformations from the system’s phase space. The idea behind this approach is to better the accuracy of the free energy calculations by capturing the variance in the system’s behavior over time and across various energetic levels.

The entropy contribution was one factor that was not considered for the calculations. This choice was made solely based on the computation required to make it. The multifaceted character of entropy, which includes contributions from translational, rotational, vibrational, and structural degrees of freedom, makes it crucial to note that estimating entropy reliably and effectively in biomolecular systems is a challenging process. Additionally, studies have indicated that the improvement in results brought by the inclusion of entropy in free energy calculations can often be marginal [112]. Some research even suggests that the inclusion of entropy may detrimentally affect results by introducing unnecessary noise or inaccuracies [113,114]. Given these considerations, the decision was made to omit entropy in the current binding free energy calculations, prioritizing computational efficiency and robustness of results.

#### 4.9.4. RMSD, RMSF, and Rg

The analysis of molecular dynamics simulation trajectories for structural parameters, such as root-mean-square deviation (RMSD) and root-mean-square fluctuation (RMSF), was performed using the cpptraj module, a versatile and widely used tool for processing and analyzing trajectory data [115].

The average deviation of a collection of atoms (such as the atoms in a protein backbone) from their places in a reference structure is measured by the RMSD. In this instance, the simulation’s initial frame from the production phase served as the reference structure. RMSD can shed light on the protein structure’s overall stability throughout the simulation as well as the degree of conformational changes compared to the starting structure.

In a similar manner, RMSF was determined in relation to the first frame from the production phase. RMSF, as opposed to RMSD, offers insights on the protein’s structural dynamics at the residue level. The RMSF provides insight into the flexible and rigid portions of a protein by calculating the variation of individual residues about their average positions. Areas of the protein that experience significant conformational changes correspond to regions of high RMSF, and vice versa.

## 5. Conclusions

Several natural chalcone-type compounds have been identified in plants. Among this group, compounds with the greatest anti-cancer activity include hydroxy-methoxylated chalcones. From the inflorescences of the *Chromolaena tacotana*, we have isolated a new chalcone identified as 2′,3,4-trihydroxy-4′,6′-dimethoxychalcone. This compound exhibits significant antiproliferative activity against cancer cells, particularly TNBC. It induces both autophagy by affecting the structural conformation of mTOR protein and apoptosis dependent on caspases 3/7 activation. Furthermore, it affects mitochondrial membrane potential and downregulates anti-apoptotic Bcl-2 members, thus activating the intrinsic pathway. The study provides evidence of the potential of *Chromolaena tacotana* as an anti-cancer agent, due to its high flavonoid content. Further investigation of 2′,3,4-trihydroxy-4′,6′-dimethoxychalcone and other compounds from the plant in both in vitro and in vivo assays is warranted.

## Figures and Tables

**Table 2:** ^1^H and ^13^C NMR (300 MHz, acetone-d_6_) spectral data for the chalcone obtained from inflorescences of *Ch. tacotana*.

Position	Chalcone	
	δH (*J* in Hz)	δC (ppm)
C-1	-	127.73
C-2	7.27 _(1H, d, *J =* 2.1 Hz)_	114.44
C-3	-	145.48
C-4	-	148.11
C-5	6.91_(1H, d, *J* = 8.2 Hz)_	115.61
C-6	7.14 _(1H, dd, *J* = 8.3, 1.9 Hz)_	122.33
C-α	7.71 _(1H, d, *J* = 15.5 Hz)_	124.20
C-β	7.86 _(1H, d, *J* = 15.5 Hz)_	142.21
C-β′	-	192.42
C-1′	-	105.92
C-2′	-	166.36
C-3′	6.10 _(1H, d, *J* = 2.4 Hz)_	93.78
C-4′	-	168.26
C-4′-OCH_3_	3.89 _(3H, s)_	55.17
C-5′	6.13 _(1H, d, *J* = 2.4 Hz)_	90.90
C-6′	-	162.77
C-6′-OCH_3_	4.02 _(3H, s)_	55.62

**Table 3:** UV spectral data (in nm) for the chalcone obtained from inflorescences of *Ch. tacotana*.

Reactive	Chalcone		
	Band II (nm)	Band I (nm)	Displacement (nm)
MeOH	276	387	-
MeOH + MeONa	276	479	B I:72
MeOH + AcONa	276	417	B I: 30
MeOH + AcONa + H_3_BO_3_	276	415	B I:28
MeOH + AlCl_3_	276	401	B I:14
MeOH + AlCl_3_ + HCl	276	387	NCh.

## Data Availability

The data presented in this study supporting the results are available in the main text and Supplementary Material. Additional data are available upon reasonable request from the corresponding author.

## Supplementary Materials

The following supporting information can be downloaded at: https://www.mdpi.com/article/10.3390/ijms242015185/s1.
