# Supplementary material for: A Novel Tri-Hydroxy-Methylated Chalcone Isolated from Chromolaena tacotana with Anti-Cancer Potential Targeting Pro-Survival Proteins
Source: Int J Mol Sci. 2023 Oct 14;24(20):15185. doi: 10.3390/ijms242015185 (PMC10607159; doi:10.3390/ijms242015185)
Supplement: Supplementary file 1 [file ijms-24-15185-s001.zip › ijms-2622867-supplementary.pdf]

SUPPLEMENTARY INFORMATION

A

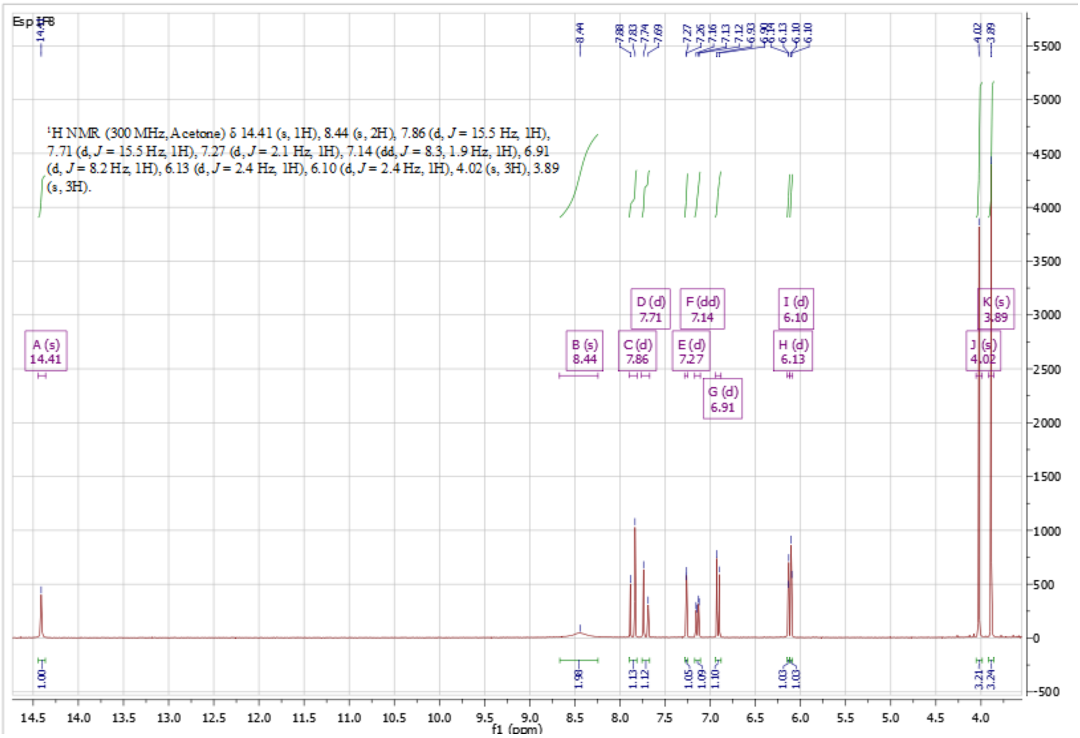

B

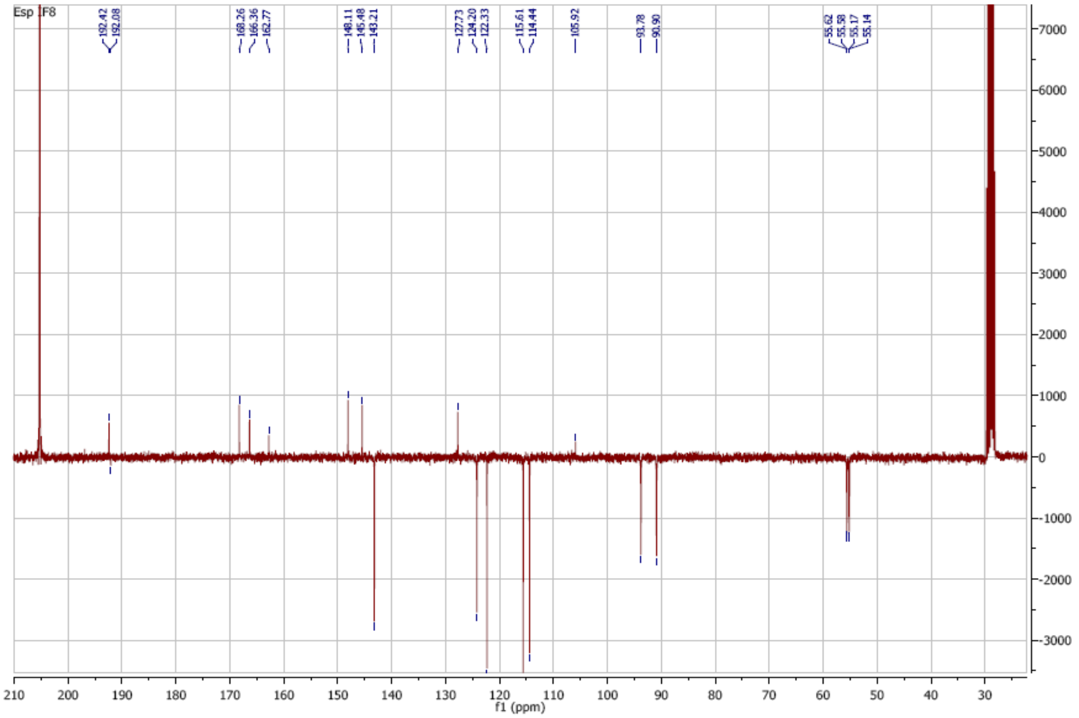

Figure S1: Nuclear Magnetic Resonance Spectroscopy of Chalcotanina (2′,3,4-trihidroxy- 4′,6′-dimetoxichalcone). A. ^{1}H NMR spectrum. B. ^{13}C NMR spectrum (75 MHz, $CDCl_3$)

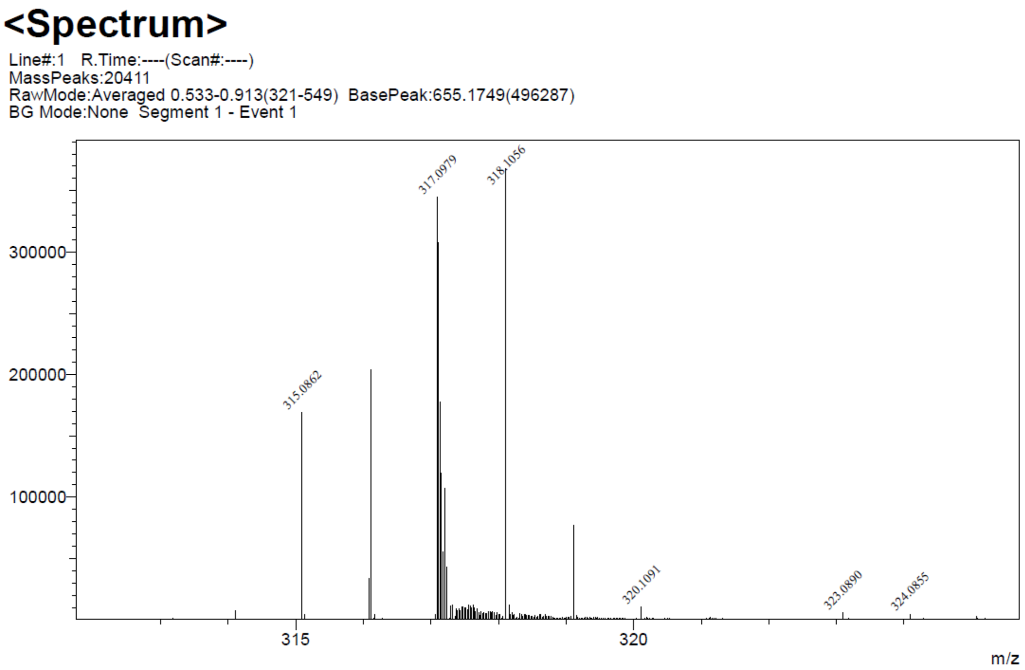

Figure S2. HPLC/QTOF analysis ESI positive ion mode of Chalcotanina $[M+H]^+$: 317,00979

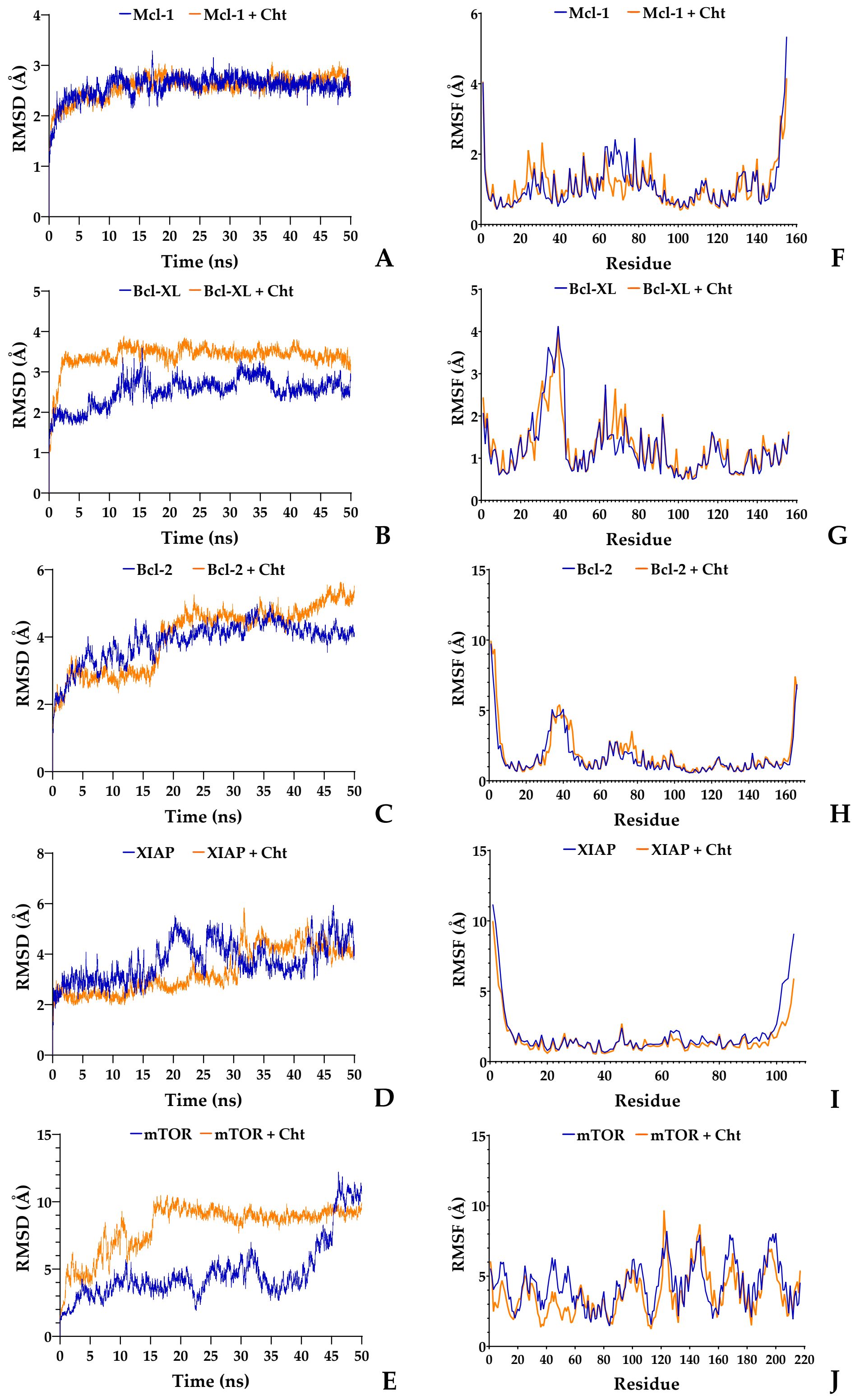

Figure S3. The root-mean-square deviation and fluctuation of pro-survival proteins in response to Chalcotanina. (A-D) RMSD trajectories for each protein: (A) Bcl-2, (B) Bcl-XL, (C) Mcl-1, (D) mTOR,

(E) XIAP. Blue traces represent the standalone protein, while the orange traces depict the protein-ligand complex with Chalcotanina. (F-J) RMSF profiles for each protein: (F) Mcl-1, (G) Bcl-XL, (H) Bcl-2, (I) XIAP and (J) mTOR. The blue shades indicate the protein in its unliganded state, while the orange traces represent the bound state with Chalcotanina.
